# Noninvasive electrocardiographic assessment of ventricular activation and remodeling response to cardiac resynchronization therapy

**DOI:** 10.1016/j.hroo.2021.01.004

**Published:** 2021-01-12

**Authors:** Thomas Jackson, Simon Claridge, Jonathan Behar, Cheng Yao, Mark Elliott, Vishal Mehta, Justin Gould, Baldeep Sidhu, Helder Pereira, Steven Niederer, Gerald Carr-White, Christopher A. Rinaldi

**Affiliations:** ∗ School of Biomedical Engineering and Imaging Sciences, King’s College London, London, United Kingdom; † Medtronic Ltd./CardioInsight, Cleveland, Ohio; ‡ Guy’s and St Thomas’ NHS Trust, London, United Kingdom

**Keywords:** Body surface mapping, Cardiac resynchronization therapy, ECG imaging, Heart failure, LV activation mapping

## Abstract

**Background:**

Cardiac resynchronization therapy (CRT) produces acute changes in electric resynchronization that can be measured noninvasively with electrocardiographic body surface mapping (ECGi). The relation between baseline acute electrophysiology metrics and their manipulation with CRT and reverse remodeling is unclear.

**Objective:**

To test (ECGi) derived parameters of electrical activation as predictors of volumetric response to CRT.

**Methods:**

ECGi was performed in 21 patients directly following CRT implant. Activation parameters (left ventricular total activation time [LVtat], global biventricular total activation time [VVtat], global left/right ventricular electrical synchrony [VVsync], and global left ventricular dispersion of activation times [LVdisp]) were measured at baseline and following echocardiographically optimized CRT. Remodeling response (>15% reduction left ventricular end-systolic volume) was assessed 6 months post CRT.

**Results:**

Patients were aged 68.9 ± 12.1 years, 81% were male, and 57% were ischemic. Baseline measures of dyssynchrony were more pronounced in left bundle branch block (LBBB) vs non-LBBB. ECGi demonstrated a trend of greater interventricular dyssynchrony between responders and nonresponders that did not reach statistical significance (VVsync: -45.7 ± 22.4 ms vs -25.1 ± 29.3 ms, *P* = .227). Remaining activation parameters were similar between responders and nonresponders (VVtat 101 ± 22.0 ms vs 98.9 ± 23.4 ms, *P* = .838; LVtat 86.4 ± 17.1 ms vs 85.1 ± 27.7 ms, *P* = .904; LVdisp 28.2 ± 6.3 ms vs 27.0 ± 8.7 ms, *P* = .726). In volumetric responders activation parameters were significantly improved with CRT compared to nonresponders: VV sync (-45.67 ± 22.41 ms vs 2.33±18.87 ms, *P* = .001), VVtat (101 ± 22.04 ms vs 71 ± 14.01 ms, *P* = .002), LVtat (86.44 ± 17.15 ms vs 67.67 ± 11.31 ms, *P* = .006), and LVdisp (28.22 ± 6.3 ms vs 21.56 ± 4.45 ms, *P* = .008).

**Conclusion:**

Baseline ECGi activation times did not predict CRT volumetric response. Volumetric responders exhibited significant improvements in ECGi-derived metrics with CRT. ECGi does not select CRT candidates but may be a useful adjunct to guide left ventricle lead implants and to perform postimplant CRT optimization.

## Graphical abstract

**Figure:**
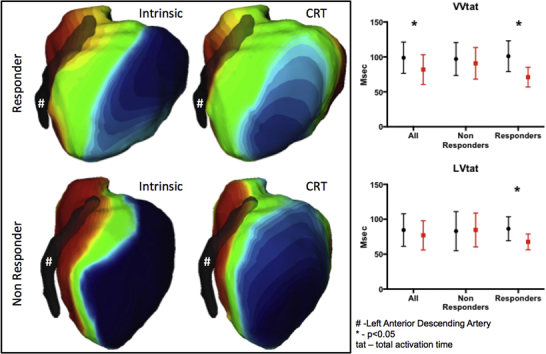

## Introduction

Current guidelines for cardiac resynchronization therapy (CRT) patient selection rely upon metrics of electrical dyssynchrony derived from the surface 12-lead electrocardiogram (ECG), with left bundle branch block (LBBB) being a marker of favorable response.^1^^,^^2^ Despite this, clinical response remains unpredictable, with up to 50% of class IIa indicated patients not responding,^3^ and echocardiographic measures to improve/predict CRT response have poorly reproducibility when evaluated systematically in multiple centers.^4^ Techniques that accurately characterize underlying CRT-amenable patterns of dyssynchrony, such as total activation times and measures of intra- and interventricular electrical dyssynchrony, may therefore offer promise with electrocardiographic body surface mapping (ECGi), a noninvasive high-fidelity technique that permits generation of activation maps and timings, being proposed as such a technique.^5^ Prior single-center studies have proposed that ECGi metrics of baseline dyssynchrony have greater predictive accuracy than the surface ECG for both symptomatic CRT response^5^ and acute contractility changes (dP/dt_max_).^6^ In addition to baseline measures of dyssynchrony, acute changes in ventricular activation with CRT can be accurately assessed with ECGi. The relation between acute changes in ventricular activation metrics and longer-term changes in terms of volumetric remodeling is unclear.

We hypothesized that acute changes in ventricular activation would predict improved volumetric response following CRT. We investigated the validity of ECGi to characterize baseline activation measures in CRT recipients and to assess acute changes in activation parameters following CRT in relation to volumetric remodeling response.

## Methods

### Study population and investigations

The local ethics authority approved the study and all patients provided written informed consent; the study complies with the Declaration of Helsinki (ClinicalTrials.gov Identifier: NCT01831518). Consecutive patients at St Thomas’ Hospital with guideline-indicated criteria for CRT (NYHA II–IV heart failure, ejection fraction ≤35%, QRS duration ≥120 ms) were prospectively included in the study.

### CRT implant and ECGi mapping

Transvenous biventricular pacing was undertaken using standard techniques. Positioning of the left ventricular (LV) and right ventricular (RV) leads were at the operators’ discretion and dependent upon target vessels and optimal electrical parameters with empirical placement of the LV lead targeted to a posterolateral vein. Biventricular pacing was not activated directly after implantation. ECGi was undertaken the following day prior to discharge. The device was turned on as part of the ECGi protocol and echocardiographic optimization of the atrioventricular and ventriculo-ventricular delays was performed using the iterative mitral valve inflow and LV outflow tract velocity time integral methods to achieve standardized optimal biventricular pacing.^7^ Baseline and CRT-on activation maps were acquired at this point using a 252-electrode high-resolution ECG mapping system (ecVUE; CardioInsight Technologies Inc, Cleveland, OH). Body surface potentials were collected from the 252 electrodes positioned around the thorax supported in a single-use vest; following this, a thoracic computed tomography (CT) scan was performed in order to orientate each electrode to the epicardial shell. Subsequent to segmentation of the cardiac silhouette and the electrode positions from the thoracic CT images, 1500 unipolar electrograms were reconstructed as previously described.^8^ Good intraobserver reproducibility has previously been demonstrated in CRT patients with the CardioInsight system (intraclass correlation coefficients of 0.92–0.99).^5^ Patients underwent standard transthoracic echocardiographic assessment. Analysis was performed with Echo Pac version 6.0.1 (General Electric-Vingmed, Horten, Norway) providing data for LV function and volumes before implantation and at 6 months follow-up. Ejection fraction and LV dimensions were measured using the 2-dimensional modified biplane Simpson method. End-systolic volume (ESV) was recorded before implantation and at 6 months. Reverse remodeling was defined as a reduction in an ESV of ≥15%.^9^

### Data analysis

The raw epicardial mapping data of intrinsic activation and echo-optimized biventricular pacing activation was analyzed with ecSYNC software (Supplemental Material; CardioInsight Technologies Inc, Cleveland, OH).

Four ventricular activation metrics were calculated:

(1) Global biventricular total activation time (VVtat), defined as the maximum of (mean of maximum 10% of LV activation times and mean of maximum 10% of RV activation times) minus the minimum of (mean of minimum 10% of LV activation times and mean of minimum 10% of RV activation times);
(2) LV total activation time (LVtat), defined as the average of the maximum 10% of activation times in the left ventricle minus the average of the minimum 10% of activation times;
(3) Global left/right ventricular electrical synchrony (VVsync), defined as the mean activation time in the right ventricle minus the mean activation time in the left ventricle;
(4) Global LV dispersion of activation (LVdisp) calculated as the standard deviation of the activation times in the left ventricle, defined as the standard deviation of activation times in the LV region of interest.

These metrics were assessed at baseline and following acute CRT. Remodeling response was assessed at 6 months and changes in electrical parameters were compared on the basis of volumetric response.

### Statistical analysis

Statistical analysis was performed with PASW Statistics version 24 software (SPSS Inc, Chicago, IL). Group comparisons were performed using an independent-samples *t* test or a paired-samples *t* test for normally distributed data. Nonparametric data were compared using the Mann-Whitney *U* test. Categorical variables were compared using the Fisher exact test or the χ^2^ test dependent on number. Receiver operator characteristic (ROC) curve analysis was used to define potential cut-offs for predictors of response. Values of *P* < .05 were considered statistically significant.

## Results

### Baseline characteristics

Twenty-one consecutive patients were prospectively studied, with a mean age of 68.9 ± 12.1 years; 17 (81%) were male. The predominant etiology was ischemic (12/21, 57%), and the majority of patients were NYHA class III (86%). Mean preimplant LV ejection fraction was 25.1% ± 8.2%. Mean QRS duration was 165 ± 21 ms. QRS morphology was LBBB in 10 (48%), with 9 of these patients having strict LBBB^10^; 6 were RV paced (28%); 1 had right bundle branch block (5%); and 4 had nonspecific interventricular conduction delay (19%). One patient had an LV lead displacement directly post implant; therefore baseline activation data could be acquired, but biventricular pacing activation parameters were unable to be measured and thus were not included in the analysis of activation changes with CRT.

### Baseline measures of electrical activation and volumetric response

The mean baseline activation parameters (Table 1) for the entire group were as follows: VVsync -33.9 ± 27.9 ms, VVtat 99.8 ± 22.3 ms, LVtat 85.7 ± 23.2 ms, and LVdisp 27.5 ± 7.6 ms. Patients with and without LBBB had similar surface ECG QRS duration (164 ± 19 ms vs 167 ± 22 ms, respectively, *P* = .79). Despite this, ECGi demonstrated a greater degree of LV/RV dyssynchrony with LBBB (VVsync in patients with LBBB vs without: -51.0 ± 19.0 ms vs -18.4 ± 26.0 ms, *P* = .004), reflecting a greater degree of ventricular electrical uncoupling despite a similar QRS duration. Comparison between strict LBBB, nonspecific interventricular conduction delay, and RV pacing exaggerated this difference between VVsync (-53.22 ± 18.75 ms vs -30.50 ± 16.94 ms vs -18.33 ± 24.45 ms, respectively, *P* = .006).

**Table 1:** Baseline activation parameters of all patients and when separating by presence or absence of left bundle branch block and with further comparison between strict left bundle branch block, nonspecific conduction delay, and right ventricular pacing

	All	LBBB	Not LBBB	*P*
QRS duration	165 ± 21	164 ± 19	167 ± 22	.79
VVsync	-33.9 ± 27.9	-51.0 ± 19	-18.4 ± 26	.004
VVtat	99.8 ± 22.3	108.1 ± 22.0	92.3 ± 20.6	.11
LVtat	85.7 ± 23.2	91.1 ± 21.8	80.8 ± 24.4	.32
LVdisp	27.5 ± 7.6	28.8 ± 7.5	26.4 ± 7.9	.48

	Strict LBBB	NICD	RV Paced	*P*
QRS duration	168.11 ± 15.50	149.00 ± 24.14	177.33 ± 14.68	.06
VVsync	-53.22 ± 18.75	-30.50 ± 16.94	-18.33 ± 24.45	.006
VVtat	112.11 ± 19.05	82.25 ± 23.20	98.00 ± 20.06	.13
LVtat	95.67 ± 17.36	78.00 ± 18.39	83.67 ± 31.08	.34
LVdisp	30.33 ± 6.08	24.75 ± 4.57	27.50 ± 10.45	.37

LBBB = left bundle branch block; LVdisp = global left ventricular dispersion of activation; LVtat = left ventricular total activation time; NICD = nonspecific interventricular conduction delay; RV = right ventricular; VVsync = global left/right ventricular electrical synchrony; VVtat = global biventricular total activation time.

**Figure 1:**
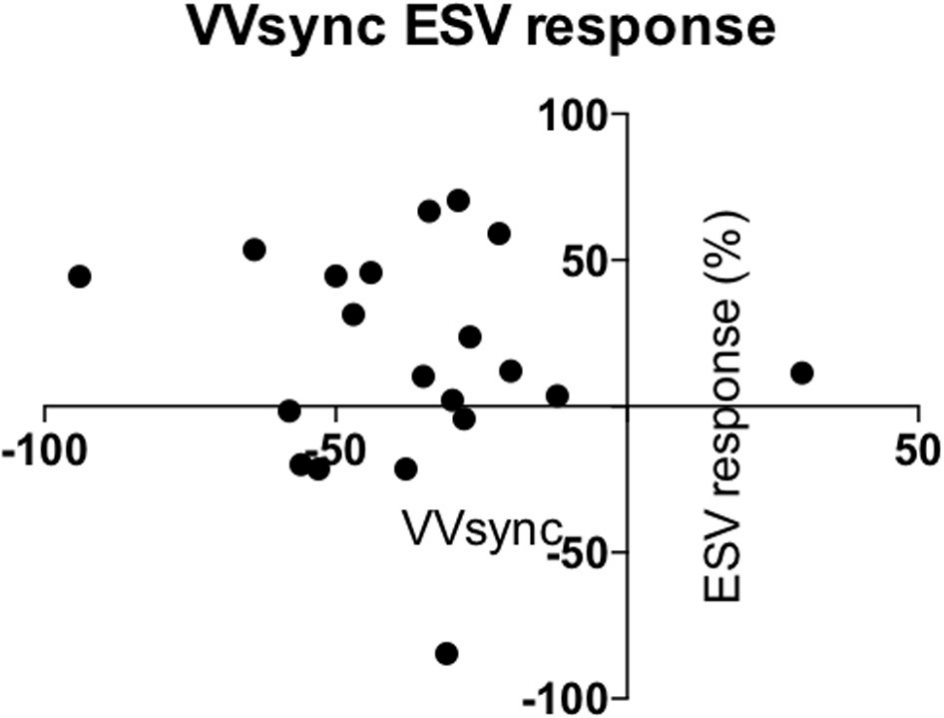
Scatter diagram of change in end-systolic volume (ESV) following cardiac resynchronization and global left/right ventricular electrical synchrony (VVsync).

In total there were 9 (43%) patients who remodeled with CRT, including all 4 female patients (*P* = .021) (Table 2). Baseline QRS duration did not differentiate volumetric responders from nonresponders (165 ± 20 ms vs 166 ± 21 ms, *P* = .897). ECGi demonstrated a trend to a greater degree of interventricular dyssynchrony between responders and nonresponders that did not reach statistical significance (VVsync: -45.7 ± 22.4 ms vs -25.1 ± 29.3 ms, *P* = .227). The other baseline activation parameters were similar between responders and nonresponders (VVtat 101 ± 22.0 ms vs 98.9 ± 23.4 ms, *P* = .838; LVtat 86.4 ± 17.1 ms vs 85.1 ± 27.7 ms, *P* = .904; LVdisp 28.2 ± 6.3 ms vs 27.0 ± 8.7 ms, *P* = .726) (Table 2). ROC curve analysis corroborated this, with the area under the curve (AUC) for each parameter varying between 0.514 (LVdisp) and 0.657 (VVsync).There was no correlation between VVsync and volumetric response (r = -0.11, *P* = .640) (Figure 1). There was no difference in baseline activation parameters between women and men (VVsync -50.0 ± 31.5 ms vs -31.1 ± 26.6 ms, *P* = .208; VVtat 102.5 ± 29.9 ms vs 99.2 ± 21.2 ms, *P* = .796; LVtat 87.5 ± 18.0 ms vs 85.3 ± 24.8 ms, *P* = .869; LVdisp 27.8 ± 5.9 ms vs 27.5 ± 8.1 ms, *P* = .949).

**Table 2:** Preimplant demographics and baseline activation parameters in volumetric responders and nonresponders

	Responder	Nonresponder	*P*
Sex			
M	5	12	.021
F	4	0	
LBBB			
Yes	5	5	.658
No	4	7	
QRS duration (ms)	165 ± 20	166 ± 21	.897
EF	26.7 ± 8.4	24.0 ± 8.2	.473
VVsync	-45.7 ± 22.4	-25.1 ± 29.3	.227†
VVtat	101 ± 22.0	98.9 ± 23.4	.838
LVtat	86.4 ± 17.1	85.1 ± 27.7	.904
LVdisp	28.2 ± 6.3	27.0 ± 8.7	.726

EF = ejection fraction; LBBB = left bundle branch block; LVdisp = global left ventricular dispersion of activation; LVtat = left ventricular total activation time; VVsync = global left/right ventricular electrical synchrony; VVtat = global biventricular total activation time.

† Nonparametric analysis.

### Acute changes in activation parameters with CRT and volumetric response

**Figure 2:**
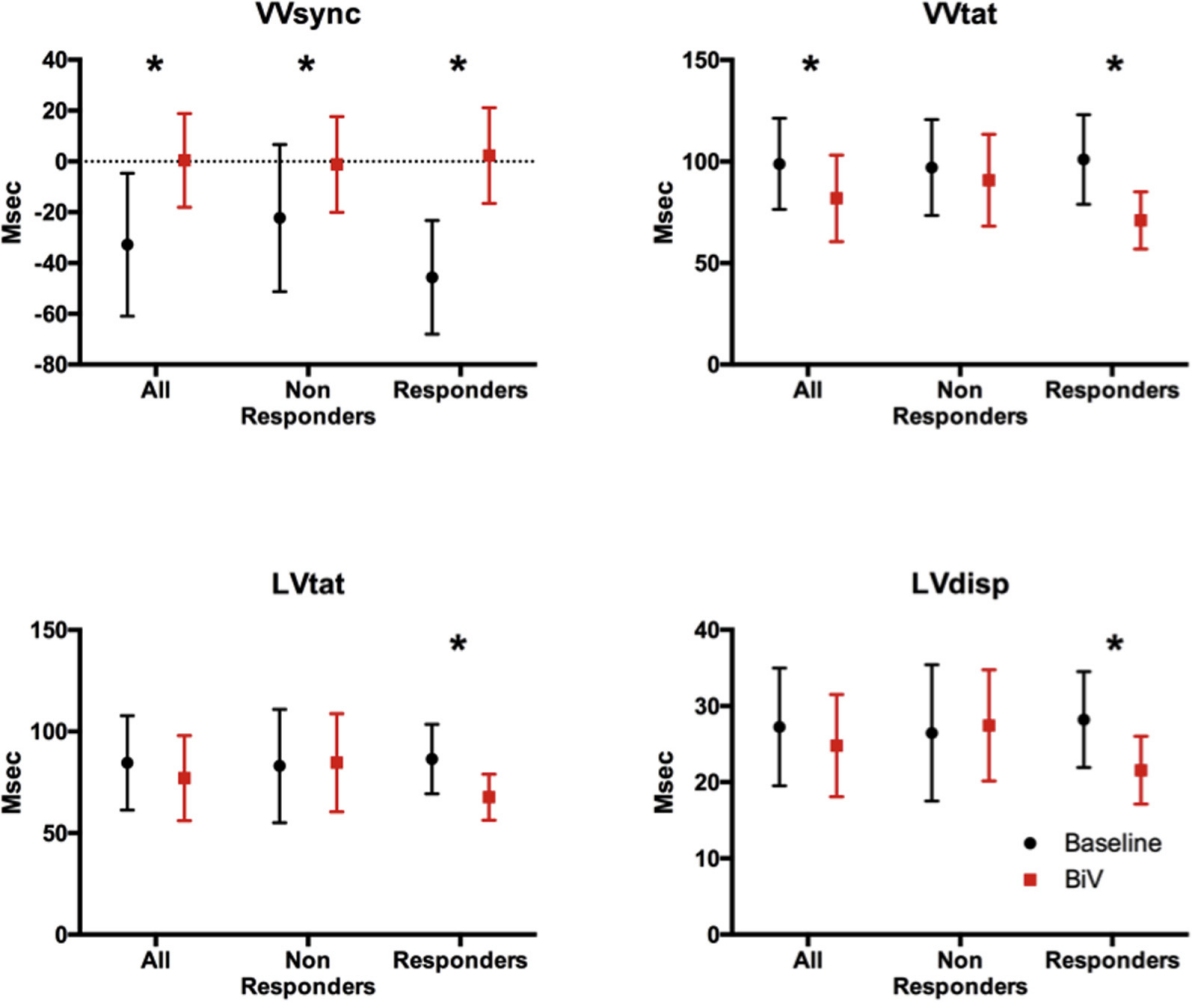
Activation time changes with echo-optimized biventricular pacing (BiV) from baseline in all patients, responders and nonresponders. ∗ indicates statistically significant. LVdisp = global left ventricular dispersion of activation; LVtat = left ventricular total activation time; VVsync = global left/right ventricular electrical synchrony; VVtat = global biventricular total activation time.

**Figure 3:**
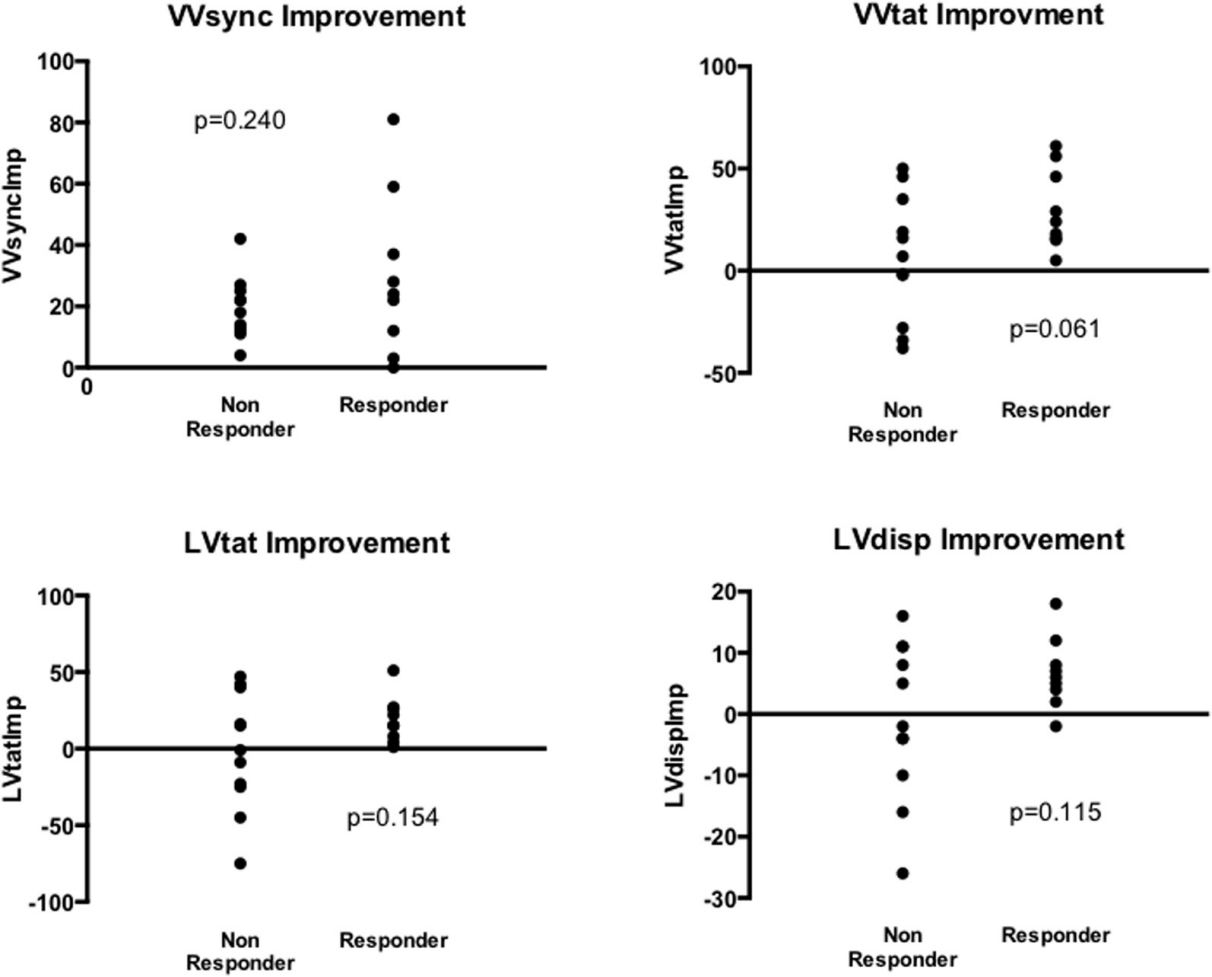
Improvement (LVdispImp, LVtatImp, VVsyncImp, VVtatImp) in activation times with cardiac resynchronization therapy from baseline, dichotomized by responders and nonresponders. Units are ms. Abbreviations as in Figure 2.

Changes in activation time were calculated at baseline and post echocardiography-optimized biventricular pacing in responders and in nonresponders. For the entire group, echocardiography-optimized biventricular pacing resulted in acute reductions in activation times and improvement in interventricular dyssynchrony from baseline. VVsync was significantly improved with CRT (-32.80 ± 28.19 ms vs 0.40 ± 18.45 ms, *P* < .001) and VVtat was significantly shortened with CRT (98.85 ± 22.40 ms vs 81.9 ± 21.30 ms, *P* = .015). When patients were dichotomized into volumetric responders and nonresponders there was a difference in the magnitude of acute activation time changes with CRT. In volumetric responders all activation parameters were significantly reduced with CRT: VV sync (-45.67 ± 22.41 ms vs 2.33 ± 18.87 ms, *P* = .001), VVtat (101 ± 22.04 ms vs 71 ± 14.01 ms, *P* = .002), LVtat (86.44 ± 17.15 ms vs 67.67 ± 11.31 ms, *P* = .006), and LVdisp (28.22 ± 6.3 ms vs 21.56 ± 4.45 ms, *P* = .008). In nonresponders only VVsync improved, whereas the rest of the parameters were not improved with CRT: VV sync (-22.27 ± 28.94 ms vs -1.18 ± 18.86 ms, *P* = .037), VVtat (97.09 ± 23.61 ms vs 90.82 ± 22.59 ms, *P* = .514), LVtat (83.00 ± 27.96 ms vs 84.64 ± 24.20 ms, *P* = .891), and LVdisp (26.45 ± 8.95 ms vs 27.45 ± 7.29 ms, *P* = .801) (Table 3, Figure 2). Relative activation time improvements were calculated; for VVsync this was calculated as the improvement towards zero. There were no significant differences between these results between responders and nonresponders; however, improvement of VVtat (VVtatImp) with biventricular pacing approached significance (30.0 ± 19.7 ms vs 6.3 ± 30.8 ms, *P* = .061). All responders had improvement with VVtat and LVtat with CRT and all but 1 responder had improvements in LVdisp; these improvements were not seen in all nonresponders (Figure 3). ROC analysis generated an AUC for VVtatImp of 0.727 (VVsyncImp 0.601, LVtatImp 0.667, LVdispImp 0.672), with a VVtatImp > 11 ms in the group having a sensitivity of 0.889 and a specificity of 0.545 (Table 4).

**Table 3:** Changes in activation times from baseline to echo-optimized biventricular pacing for all patients, nonresponders and responders

	Baseline (ms)	Echo-optimized BiV pacing (ms)	*P* value
All			
VVsync	-32.8 ± 28.19	0.40 ± 18.45	<.001
VVtat	98.85 ± 22.40	81.90 ± 21.30	.015
LVtat	84.55 ± 23.20	77.00 ± 20.91	.150†
LVdisp	27.25 ± 7.725	24.80 ± 6.732	.321
Nonresponders			
VVsync	-22.27 ± 28.94	-1.18 ± 18.86	.037
VVtat	97.09 ± 23.61	90.82 ± 22.59	.514
LVtat	83.00 ± 27.96	84.64 ± 24.20	.891
LVdisp	26.45 ± 8.95	27.45 ± 7.29	.801
Responders			
VVsync	-45.67 ± 22.41	2.33 ± 18.87	.001
VVtat	101.00 ± 22.04	71.00 ± 14.01	.002
LVtat	86.44 ± 17.15	67.67 ± 11.31	.006
LVdisp	28.22 ± 6.30	21.56 ± 4.45	.008

LVdisp = global left ventricular dispersion of activation; LVtat = left ventricular total activation time; VVsync = global left/right ventricular electrical synchrony; VVtat = global biventricular total activation time.

Note 1 patient was excluded from this analysis owing to left ventricular lead displacement post implant.

† Nonparametric analysis.

**Table 4:** Improvement with activation times between baseline (intrinsic) activation and echo-optimized biventricular pacing compared between volumetric responders and nonresponders

	All	Responder	Nonresponder	*P*
VVsyncImp	23.8 ± 19.4	29.6 ± 26.3	19.1 ± 10.2	.240
VVtatImp	17.0 ± 28.4	30.0 ± 19.7	6.3 ± 30.8	.061
LVtatImp	7.6 ± 31.5	18.8 ± 15.2	-1.6 ± 38.6	.154
LVdispImp	2.5 ± 10.8	6.7 ± 5.8	-1.0 ± 12.8	.115

LVdispImp = improvement of global left ventricular dispersion of activation; LVtatImp = improvement of left ventricular total activation time; VVsyncImp = improvement of global left/right ventricular electrical synchrony; VVtatImp = improvement of global biventricular total activation time.

## Discussion

We assessed the baseline values and acute changes following CRT of 4 ventricular activation metrics derived from ECGi.

The main findings are as follows: (1) Baseline activation metrics demonstrated greater interventricular dyssynchrony (ventricular electrical uncoupling) in LBBB patients compared to other intrinsic QRS morphologies despite similar QRS duration, suggesting ECGi provides useful information regarding ventricular activation in addition to surface ECG–derived QRS duration and morphology. (2) Baseline activation metrics did not reliably predict CRT volumetric response. There was a signal for greater interventricular dyssynchrony, which did not reach statistical significance, but no reliable correlation between the degree of interventricular dyssynchrony and the magnitude of volumetric response. (3) There were significant improvements in ECGi-measured activation timings with optimized biventricular pacing compared to baseline in volumetric CRT responders, which were not apparent in nonresponders.

### Baseline activation timings and CRT response

Longer ventricular activation derived from the 12-lead ECG is known to predict a greater degree of response to CRT^11^ and is reflected by an increase in the recommended QRS duration from 120 to 130 ms in the current European guidelines.^12^ ECGi timings have been previously correlated with surface 12-lead ECG timings and more accurately represent epicardial electrophysiology, as they take into account differences in individual patient anatomy and the effect this has on the surface electrical signal.^13^ In the current study, baseline activation parameters did not reliably predict volumetric CRT response. Our findings are in contradiction to the findings of Ploux and colleagues^5^ that demonstrated that ventricular electrical uncoupling (VEU), a measure reflecting LV/RV dyssynchrony and equivalent to VVsync, predicted *clinical* response to biventricular pacing in 33 consecutive CRT patients from a single center (AUC of 0.72, *P* = .033), with a 50 ms cut-off for VEU having 90% sensitivity and 82% specificity.^5^ In a further study of 61 patients receiving CRT, Ploux and colleagues^6^ demonstrated a strong correlation between baseline VEU and acute change in LV dP/dt_max_ (r = 079, *P* < .001), an acute hemodynamic response parameter recognized as a predictor for ventricular remodeling.^14^ In the current study VVsync, which is an equivalent parameter to VEU (mean activation of the LV subtracted from the RV mean activation for VVsync, whereas it is RV – LV for VEU, ie, an inverse relationship), did not predict volumetric response. The nonsignificant trend in our data to a greater VVsync between responders and nonresponders is potentially attributable to the smaller size of our cohort; however, within our patients the baseline QRS duration was longer (165 ± 21 ms vs 152 ± 22 ms, *P* = .0359). Another explanation is that this metric may not be robust when tested in a different center; this is a similar effect as seen with echo-based dyssynchrony parameters and response to CRT.^4^ The relatively low level of volumetric response may reflect the higher percentage of ischemic patients in our cohort (57%, in comparison to 42% in Ploux’s clinical response population), and although such metrics may predict clinical response and acute hemodynamic response it does not predict remodeling. In support of this, “true VEU” was present in 6 (29%) of our patients; however, there was no difference in amount of remodeling between those with true VEU and those without (ESV improvement 16.6% ± 34.8% vs 16.2% ± 40.1%, *P* = .983). The lack of correlation between the VVsync and reverse remodeling is also not supportive of the use of baseline metrics of activation being able to predict volumetric response.

### Activation time improvement and response

QRS shortening after biventricular pacing is associated with volumetric response to CRT^15^; therefore, one would expect changes in ECGi-derived activation times (LVtat, VVtat, and VVsync) would also have this correlation. In keeping with this are the findings of Ploux and colleagues,^6^ who demonstrated a correlation between change in activation times from baseline with biventricular pacing and change in dP/dt_max_ and, furthermore, the concept of iatrogenic electrical dyssynchrony with worsening (prolongation) of activation times associated with a deterioration in acute hemodynamic response. Our current findings are in keeping with this, with significant improvement in activation times with CRT in patients that exhibited favorable volumetric remodeling and all patients who had acute worsening in activation times with CRT not responding; this supports the hypothesis that acute electrical resynchronization translates into favorable chronic ventricular remodeling. A non–statistically significant improvement in VVtat was associated with reverse remodeling, raising the potential of this metric in determining LV lead placement, guiding postimplant optimization, and predicting CRT response.

### Clinical perspective

Our results suggest that although intrinsic activation times are important for patient selection, they are not the only factor to deliver response. In our cohort the degree of electrical improvement with CRT from baseline appears to have more consequence than the baseline parameters themselves. Therefore, CRT optimized to improve electrical dyssynchrony, either during implant with necessary revision of the ECGi data acquisition process, at optimization, or using preprocedural simulations,^16^ could potentially transform a nonresponder into a responder. The ECG Belt Study is currently recruiting and investigates the utility of an ECGi belt at CRT implant and follow-up to enhance clinical outcomes (Clinicaltrials.gov number NCT03504020), and we have previously shown that ECGi can characterize acute changes in activation metrics with different programming of multipolar LV electrodes, suggesting that vector optimization may play a role in CRT optimization.^17^ ECGi may also have a potential role in optimization of AV and VV timings to achieve optimal electrical resynchronization, as VVtat improvement with biventricular pacing is a potentially promising tool.

### Limitations

This is a small mechanistic and hypothesis-generating study and is therefore open to bias secondary to the sample size; however, the small sample size is in keeping with routine clinical work and therefore questions the utility of body surface mapping for selection and monitoring of CRT patients in the clinical setting. In order to analyze the maps from the cardiac CT–derived epicardial shell, the border of the left ventricle is defined by annotating the left anterior descending artery as it travels down the anterior interventricular groove from base to apex. This impacts LVtat measurements and does not take into account variability in septal anatomy and inferior RV insertion lines. In this cohort LVtat estimation does not predict volumetric response and this value seems very similar between responders and nonresponders; therefore it is difficult to see how refinement in the anatomical processing will impact these predictive rates. One consideration for both LVtat and VVtat is that these measures are without direction; CRT manipulates activation wavefronts between 2 (or more) points in space, and therefore if there is a delay in intrinsic activation from apex to base where the leads are in a septal-to-inferolateral orientation then this is unlikely to impact total activation times, with corresponding low impact on remodeling response. LVdisp can be impacted by how the left ventricle is defined in postprocessing, as with LVtat, including not taking into account septal activation. The lack of difference between responders and nonresponders in LVdisp may be affected by this lack of inclusion of the septum, which is likely to be a critical shortcoming of this metric in the full assessment of LV electrical dyssynchrony.

## Conclusion

Baseline activation times from ECGi in particular electrical delays between mean RV and mean LV activation (VVsync) did not predict CRT volumetric response. This questions the clinical utility of ECGi in selection of CRT candidates. CRT volumetric responders exhibit significant improvements in ECGi-derived activation times from baseline with biventricular pacing whereas nonresponders do not, although there was no significant difference between degree of improvement between responders and nonresponders. This questions whether intraprocedural ECGi may be a useful adjunct to guide LV lead implants and to perform postimplant CRT optimization, as acute worsening in ECGi parameters indicates that a modification of CRT activation or intensification of other therapies will be needed to convert a nonresponder into a responder.

## Funding Sources

This study was supported by CardioInsight Inc and by the Wellcome/EPSRC Centre for Medical Engineering [WT203148/Z/16/Z].

## Disclosures

CY was an employee of CardioInsight. All other authors declare no conflict of interest.

## Authorship

All authors attest they meet the current ICMJE criteria for authorship.

## Patient Consent

All patients provided written informed consent.

## Ethics Statement

The study complies with the guidelines set forth in the Declaration of Helsinki. The local ethics authority approved the study.
